# Extracellular Vesicles as Therapeutic Agents in Systemic Lupus Erythematosus

**DOI:** 10.3390/ijms18040717

**Published:** 2017-03-28

**Authors:** Javier Perez-Hernandez, Josep Redon, Raquel Cortes

**Affiliations:** 1 Genomic and Genetic Diagnosis Unit, INCLIVA Biomedical Research Institute, Accesorio 4, Avd. Menendez Pelayo, 46010 Valencia, Spain; Javier.Perez-Hernandez@uv.es (J.P.-H.); josep.redon@uv.es (J.R.); 2 Research Group of Cardiometabolic and Renal Risk, INCLIVA Biomedical Research Institute, Accesorio 4, Avd. Menendez Pelayo, 46010 Valencia, Spain

**Keywords:** extracellular vesicles, exosomes, microvesicles, systemic lupus erythematosus, antigen presenting cells, autoimmunity, microRNAs, therapy

## Abstract

Systemic lupus erythematosus (SLE) is a heterogeneous autoimmune disease that affects multiple organs. Currently, therapeutic molecules present adverse side effects and are only effective in some SLE patient subgroups. Extracellular vesicles (EV), including exosomes, microvesicles and apoptotic bodies, are released by most cell types, carry nucleic acids, proteins and lipids and play a crucial role in cell-to-cell communication. EVs can stimulate or suppress the immune responses depending on the context. In SLE, EVs can work as autoadjuvants, enhance immune complex formation and maintaining inflammation state. Over the last years, EVs derived from mesenchymal stem cells and antigen presenting cells have emerged as cell-free therapeutic agents to treat autoimmune and inflammatory diseases. In this review, we summarize the current therapeutic applications of extracellular vesicles to regulate immune responses and to ameliorate disease activity in SLE and other autoimmune disorders.

## 1. Introduction

Systemic lupus erythematosus (SLE) is a chronic autoimmune disease, clinically heterogeneous, that affects different organ systems. It is characterized by the production of autoantibodies against self-antigens that form immune complex deposits [1,2]. The prevalence of SLE varies from 20 to 150 cases per 100,000 with a high prevalence in women (9:1) [3]. In addition, SLE is a relapsing and remitting disease which treatment represents a high impact on long-term medical cost [4].

SLE is a multifactorial disease in which genetic and environmental factors interact to modulate the final phenotype. Some loci have been associated to increase the risk of SLE; for example, complement components C1q and C4, major histocompatibility complex (especially the human leucocyte antigen, class II), T cell receptor and many cytokines (IL-6, IL-27, IL-12, IL-23) [5,6]. At the same time, some are typically related to other autoimmune diseases as diabetes or rheumatoid arthritis (RA) (PTPN22 and STAT4) [7]. Moreover, an epigenetic dysregulation, found in many SLE patients, has been proposed as crucial in the initiation and progression of the disease. Thus, several studies have showed altered pathways concerning DNA methylation pattern [8,9], histone acetylation [10] and microRNAs [11,12]. Furthermore, environmental factors play an important role, showing a strong association between SLE and pesticides, Epstein-bar virus, endometriosis and even postmenopausal therapy [13,14]. In this sense, hormones may trigger autoimmune responses and modulate the alternating periods of disease flares in SLE [15].

Recently, the use of novel immunotherapies in SLE has been based on targeting biological pathways involved in oncology, transplantation and other autoimmune diseases such as RA [16]. Therefore, targeted immunotherapy includes different approaches as B-cell depletion/survival (Rituximab, Bortezomib), anti-cytokine therapies (Tocilizimab, secukinumab), JAK kinase inhibitors (Tofacitinib) and immune-modulating peptides (Forigerimod) [16,17].

Although survival rates and longevity have increased, current therapeutic molecules present adverse side effects and are partially effective only with some patient subgroups, such as low interferon signatures or active SLE without nephritis [18,19]. In addition, these new drugs will have a high impact on long-term medical costs associated to the disease [20]. Despite the improvement in understanding of SLE pathogenesis and the presence of more specific therapies, these are still unsatisfactory. Over the last few years, the extracellular vesicles (EV) have been described as biological essential players in several cellular processes and carriers of nucleic acids, proteins and lipids [21,22,23]. EVs are small membranous vesicles, ranging from 40 nm to 5 µm, and receive different names depending on biogenesis, composition and origin.

In this review, we summarize the therapeutic potential and mechanism of action of EVs and its components to regulate immune responses and to ameliorate disease activity in SLE and other autoimmune disorders.

## 2. Types of Extracellular Vesicles

The general term “EV” includes different types of vesicles, not homogeneous, overlapping in size and classified according to different parameters as biochemical composition, morphology, biogenesis and size [24,25] (Table 1). Exosomes are small lipid vesicles (40 to 130 nm) derived from the inward budding of endosomal compartments, accumulating in intraluminal vesicles known as multivesicular bodies. Exosomes are released to the extracellular milieu by the fusion of endosomal compartments with the plasma membrane [26]. Due to its sorting, exosomes are enriched in tetraspanins (CD9, CD63 and CD81) and other proteins involved in vesicle trafficking and signal transduction (TSG 101, RAB family) [27]. Microvesicles or microparticles (also found in literature as shedding vesicles, ectosomes or prostasomes) are larger than exosomes, from 100 to 1000 nm, differ mainly by their mechanism of generation and include all structures released by budding and fission directly from the plasma membrane [28]. Finally, apoptotic bodies are large structures, up to 5000 nm, released as the consequence of apoptosis process and are also produced by direct budding of the membrane (Table 1). EV-uptake by the target cell can be mediated via cell receptor binding, direct fusion of membranes and endocytic internalization [24].

EVs are small membranous structures composed by a lipid bilayer, released by many type of cells and secreted in several biofluids as urine, plasma, saliva, cerebrospinal fluid, synovial fluid or breast milk [27]. EVs have the ability to carry nucleic acids (RNA types, mitochondrial DNA, single or double-stranded DNA), proteins and lipids [21,22]. The analysis of its RNA profile, coding and non-coding, has shown important differences with the distribution in the producing cells [29]. Moreover, RNA species shuttled by EV maintain their function when transferred to the recipient cells, remarking an epigenetic signaling and having an important role in cell-to-cell communication [30]. Interestingly, tumor EVs carry DNA that reflects the genetic status of the tumor [22]. EV membranes are generally enriched in cholesterol, sphingomyelin, phosphatidylserine and glicosphingolipids whereas other lipids have been suggested to be involved both in biogenesis and release [31]. Moreover, the transfer of membrane components includes proteins such as receptors and ligands but also cytokines into the luminal space [24].

## 3. Extracellular Vesicles in Systemic Lupus Erythematosus

### 3.1. Extracellular Vesicles in Immune Modulation

Most autoimmune disorders are characterized by a chronic inflammatory state, so the reduction of inflammation becomes essential in order to treat patient’s condition. EVs can be involved in the development and maintenance of exacerbated immune responses by working as autoadjuvants, initiating and perpetuating autoantibody production. In this sense, platelet-derived microparticles (MPs) can form immune complexes with autoantibodies against citrullinated peptides, that perpetuate inflammation into the synovial fluid of rheumatoid arthritis (RA) patients [32,33]. Many studies have pointed out a dysregulation of apoptosis and an ineffective clearance of apoptotic bodies as a source of autoantigens in SLE, leading to the development of autoimmunity [34,35,36]. Moreover, other studies in SLE patients have demonstrated the increase of IgG-MPs in plasma compared to controls and its correlation to dsDNA antibodies [37,38]. Complementary, EVs can also transport and transfer a broad range of cytokines and chemokines, inducing and maintaining inflammation. Thus, Lee and collaborators have reported that serum exosomes isolated from SLE patients were able to induce high cytokine production in healthy peripheral blood mononuclear cells. Interestingly, this proinflammatory response disappeared when exosome preparations were disrupted mechanically [39]. Moreover, platelet-MPs seem to release IL-1β, which promotes joint inflammation by augmenting IL-6 and IL-8 levels in fibroblasts from RA patients [40].

Recently, some studies have shown the relationship between pathogen and damage-associated molecular patterns (PAMPs and DAMPs) and EV transport. These molecules are found normally inside cells but may trigger abnormal immune responses after cellular stress, infection or injury conditions [41]. Therefore, EVs are likely to participate actively in the persistence of inflammation by promoting activation of lymphocytes and the release of pro-inflammatory cytokines. Thus, EVs are released after injury or stress and can carry nuclear proteins as HMGB1 or S100 proteins (group of ligands of toll-like receptors) leading to pro-inflammatory cytokine release [34,42]. At the same time, EVs can also act as PAMPs themselves after pathogen infection, being recognized by antigen-presenting cells [43].

On the other hand, under natural circumstances there is also a release of immunosuppressive EVs, which are also involved in the maintenance of immunological tolerance. For instance, trophoblast-derived EVs have been shown to ameliorate severity in multiple sclerosis and RA patients [44]. In addition, exposure to specific antigens has stimulated EV production with anti-allergic properties in bronchoalveolar lavage fluid [45]. Interestingly, Ostman and collaborators showed how intestinal epithelial cells were able to secrete exosome-like structures named “tolerosomes”, and expressing major histocompatibility complex proteins (MHC), able to induce food antigen tolerance in the gastrointestinal tract [46].

### 3.2. Extracellular Vesicles as Biomarkers of Systemic Lupus Erythematosus

Extracellular vesicles have been reported as reliable biomarkers of activity disease besides their role in regulating immune responses, offering a valuable complement to classical laboratory markers [47]. In this regard, circulating MP of SLE patients have been associated with clinical features suggesting an important role in driving the activation of dendritic cells and pathological responses [48,49,50]. Thus, novel subpopulations of platelet, endothelial and leukocyte-derived MP have revealed specific MP signature that could become a diagnostic and prognostic tool. On this subject, Nielsen et al. showed a correlation between a subset of MP from endothelial origin with disease activity, glomerulonephritis and vascular dysfunction [48]. In a similar way, another study has shown an increase of endothelial MP with active SLE when compared to controls, and immunosuppressive therapy reduced the cardiovascular risk by decreasing the number of circulating endothelial MP [51]. Likewise, another study has shown an increment of plasma levels of monocytic CD 14+ MP in active patients and a tight correlation with SLE disease activity [52]. Interestingly, circulating apoptotic MP in plasma presented high proinflammatory effects by stimulating cytokine release (IL-6, TNF and INF-α) in some subsets of dendritic cells. Alternatively, MP from healthy subjects or other autoimmune diseases did not show the same response [53].

In SLE, immunocomplex (IC) deposition is a key and early event in the glomerulus of Lupus Nephritis (LN) patients. Kidney affectation constitutes a major cause of morbidity and mortality, progressing to end-stage renal failure in 10–30% of SLE patients [54]. A recent study demonstrated the role of platelet-derived MP in the formation of IC by harboring IgG at their surface and also a correlation between those MP and disease activity [55]. Similarly, Nielsen et al. reported an association of the increment of circulating MP with high content of galectin-3-binding protein (G3BP) and disease activation [56]. They also proposed that the possibility of targeting MP surface molecules as G3BP may result in the reduction of IC and inflammation by decreasing the amount of accessible extracellular autoantigens [57].

On the other hand, EVs transport microRNAs that can be used as cell-free biomarkers [58]. In this sense, exosomes in urine constitute an interesting approach to study renal and urogenital diseases due to their easy access and non-invasive collection [59,60]. Furthermore, characterization of exosomal miRNAs compared to intracellular miRNAs by next-generation sequencing has confirmed urinary exosomes as a stable source of miRNA biomarkers [61]. In this sense, some studies have demonstrated changes in the amount of exosomal miRNAs in urine from LN patients. Concretely, miR-26a was found augmented and miR-29c reduced when compared to healthy controls. Moreover, those levels showed correlation with urinary protein levels and renal fibrosis, suggesting a predictive role of podocyte injury and renal function [62,63]. Finally, our group has found much higher levels of miR-146a within urinary exosomes of LN patients when compared to controls or SLE without renal affection [58]. The discriminatory role of this miRNA becomes more interesting due to its participation in the type 1 interferon pathway [12].

In summary, the amount and phenotype of circulating MPs may be used as new biomarkers of activity and progression of SLE and could provide a new therapeutic approach.

## 4. Extracellular Vesicles as Cell-Free Therapy in Autoimmunity

### 4.1. Mesenchymal Stem Cell Derived Extracellular Vesicles

Mesenchymal stem cells (MSCs) are a population of adult multipotent cells that have the ability to differentiate into mesodermal tissues. MSCs were originally described in the bone marrow (BM-MSCs), but can be isolated from many sources such as adipose tissue, dental pulp, umbilical cord blood and placenta [64]. Due to their high proliferative potential, easy access and immunosuppressive properties, MSCs have been proposed for the treatment of diverse pathological conditions [65]. In the past few years, MSC potency in tissue reparation has been related to the secretion of bioactive components rather than cell differentiation and engraftment [66]. Besides the role of classical soluble factors in the paracrine action such as cytokines and growing factors, extracellular vesicles derived from MSCs (MSC-EVs) have emerged as major components of the MSC secretome [67,68,69].

Several preclinical studies have explored the MSC-EVs potential in regeneration using both, in vivo an in vitro models. These include a broad range of diseases including myocardial infarction or ischemia, acute kidney injury, fibrotic liver and neurodegenerative diseases [70,71,72]. In the context of chronic inflammation and autoimmune disorders, MSC-EVs are immunosuppressive probably due to RNA and protein transfer [73]. Therefore, they may act as immunological active agents by inducing anti-inflammatory cytokine release and also modulating Toll-like receptor signalling [74].

Regarding autoimmunity and SLE, many studies have assessed MSCs transplantation in murine models, but very few tried to use direct “conditioned medium” (based on cell-free MSCs secretome) or directly purified MSC-EVs [75]. In a model of multiple sclerosis, MSC-microvesicles were responsible to inhibit auto-reactive lymphocyte proliferation and also stimulate the secretion of anti-inflammatory cytokines, IL-10 and TGF-β [76]. Similarly, Liu’s team found that MSC-exosomes after cell transplantation were key to rescue bone marrow MSC function in lupus murine model. Thus, Fas receptor was transferred by exosomes, which helped recipient cells to reduce intracellular miR-29b levels and ameliorated osteopenia [77]. Another attempt of treatment showed the clinical efficacy of MSC exosomes in a patient with therapy-refractory graft-versus-host disease. After 4 months of MSC-exosome therapy, cutaneous and mucosal manifestations still ameliorated and steroid administration was reduced [78].

### 4.2. Extracellular Vesicles Derived from Professional Antigen-Presenting Cells

In the last few years, the ability of EV-derived from professional antigen-presenting cells (APC) to regulate immune responses has been studied [79]. To date, the best examples have focused on dendritic cells-derived EVs (DC-EVs) in which DCs can be cultured under specific conditions in order to alter the released EV population. Therefore, DCs treated in vitro with immunosuppressive drugs or cytokines make them able to suppress immune reactions. For instance, bone marrow derived DCs that were treated with IL-10 and IL-4 were able to reduce inflammation in collagen-induced arthritis via exosome signalling [80,81]. Thus, exosomes were shown to be as therapeutic as the parental DCs which may demonstrate DC-EVs as a good approach to treat arthritis and other autoimmune disorders [82,83].

**Figure 1:**
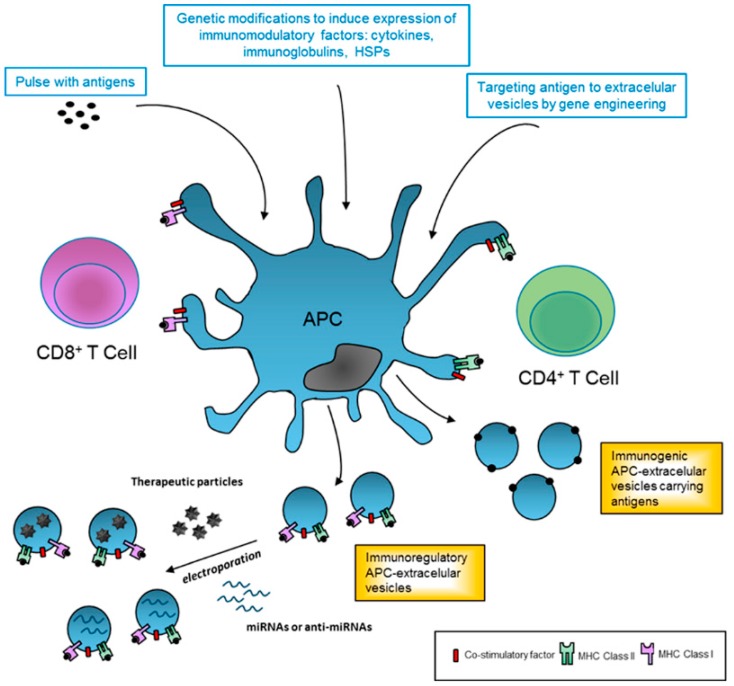
Therapeutic applications of extracellular vesicles in immune response. Professional antigen-presenting cells (APCs) can be modified in vitro in order to generate immunoregulatory or immunogenic extracellular vesicles (EVs) for therapeutic applications. In this sense, APC can be directly cultured with antigen pulses (pathogen or tumor-derived) to stimulate the release of immunogenic EVs and amplify the response. Alternatively, these modifications can also be genetically engineered to increase the levels of antigens or to express specific desired antigens in EV membranes and enhance immunogenicity. Similarly, APC can be genetically modified to express cytokines and other co-stimulatory factors that result in immunoregulatory EVs. Finally, APC-derived EVs could be modified and used as nanocarriers of pharmacological drugs or small non-coding RNAs. APC: antigen presenting cell; HSPs: heat shock proteins; IL: interleukin; MHC: major histocompatibility complex; miRNAs: microRNAs.

Furthermore, modification of APC can also be genetically engineered to improve immune regulatory EV production for therapeutics (Figure 1). In this sense, gene transfer of cytokines such as TGF-b, IL-4 or IL-10 lead to more powerful anti-inflammatory DC-EVs [82,83]. Similarly, genetic modification to express specific antigens, that are not naturally present on EVs, can enhance immunogenicity and may constitute a new type cell-free system for vaccination.

## 5. Future Research

Extracellular vesicles are key modulators of immune responses and have a vast potential as therapeutic agents for treating a variety of human disorders, including autoimmune diseases. Given their ability to modulate immune responses, EVs from MSC and APC hold a promising therapeutic approach in immune therapy. As shown in Figure 1, EVs could be safely generated and used as a tool to package small RNAs and other pharmacological and bioactive molecules to avoid immune response and enhance the therapeutic activity. In this regard, Alvarez-Erviti et al. used self-derived DCs to produce targeted exosomes (neuron-specific peptide), which were loaded with siRNA and injected into mice, where they delivered their content into the brain [84]. Similarly, patient-derived APCs could be modified in vitro to improve the immune regulation by releasing EVs with high levels of co-stimulatory ligands (Figure 1).

However, there are some issues that have to be further tested before an extensive EV implementation into the clinics. First of all, targeting EVs for a specific uptake have not been systematically evaluated [85]. For instance, mature DC-EVs are efficiently internalized by activated T cells, whereas infected B cells release EVs that bind more specifically to other B cells [86,87]. Similarly, EV uptake might not be synonym of functional delivery into the recipient cells (e.g., endosomal membrane breakdown), so quantitative analysis of EV delivery will be required for different cell types [85]. In addition, an EV-based treatment or drug is considered to be a “biological medicinal product”, so we have to discern between the “active substances” and “excipients” as well as to establish a “mode or mechanism of action” [88]. In this sense, evaluation of EV-loaded products and potency is crucial and may be challenging. Determine total protein, lipids or RNA content as well as vesicle dosage will require the development of new methods and quality control of vesicle storage and stability [85].

## 6. Conclusions

Extracellular vesicles have been recognized as key players in several cellular processes and are released by most cell types. EV-mediated horizontal transfer of nucleic acids, proteins and lipids constitutes a new paradigm of cell-to-cell communication and homeostasis. Depending on the context, EVs can stimulate or suppress the immune response. In SLE, EVs can exacerbate the immune response acting as autoadjuvants, spreading and maintaining inflammation and enhancing immunocomplex formation. The presence of EV-specific patterns and their cargo, mainly proteins and miRNAs, as biomarkers of SLE activity and progression is rapidly expanding.

The feasibility and the safety of clinically using EVs have been recently tested in autoimmune disorders. Interestingly, EVs released from MSCs mimic the beneficial effects of stem cell therapy and could be administered easily and safely to treat autoimmune and inflammatory diseases. Another cell-free strategy remarks the potential of APC-derived EVs as therapeutic agents. Therefore, autologous APC can be genetically modified to produce more immunosuppressive EVs, as well as to improve the transport of therapeutic molecules. Although further studies are necessary to guarantee an extensive application into the clinics, an EV-based therapy is expected to provide new and safe therapeutic approach to treat SLE and other autoimmune disorders.

## Figures and Tables

**Table 1:** Characteristics of extracellular vesicles.

Type and Size	Biogenesis	Markers	Contents
Exosomes (40–130 nm)	Endolysosomal pathway. Release by exocytosis of multivesicular bodies	Tetraspanins (CD63, CD9, CD81), Alix, TSG101, Hsp60, Hsp70, Hsp90	miRNA and mRNA; lipids (cholesterol, ceramide, sphingomyelin), cytokines receptors, MHC molecules
Microvesicles (100–1000 nm)	Cell surface. Outward budding of plasma membrane	Integrins, selectins, metalloproteinases, Phosphatidyl-serine	mRNA, non-coding RNAs, membrane receptors, cytoplasmic proteins (cytokines)
Apoptotic bodies (50–5000 nm)	Cell surface. Release from cellular blebs during apoptosis	Phosphatidyl-serine	Nuclear fractions, cell organelles, DNA, rRNA, mRNA

Hsp: heat shock proteins; MHC: major histocompatibility complex; mRNA: messenger RNA; miRNA: microRNA; rRNA: ribosomal RNA; TSG101: tumor susceptibility gene 101.
